# Vein-first versus artery-first ligation procedure for lung cancer surgery: An updated review

**DOI:** 10.1186/s13019-021-01658-w

**Published:** 2021-09-26

**Authors:** Tian Zhao, Chu Zhang, Chen Zhao, Wen-Bin Wu, Miao Zhang

**Affiliations:** 1 Department of Thoracic Surgery, Shanghai East Hospital, Tongji University School of Medicine, Shanghai, People’s Republic of China; 2 Department of Thoracic Surgery, Shaoxing People’s Hospital (Shaoxing Hospital, Zhejiang University School of Medicine), Shaoxing, People’s Republic of China; 3 Department of Thoracic Surgery, Xuzhou Central Hospital, 199 Jiefang South Road, Xuzhou, 221009 China

**Keywords:** Lung cancer, Circulating tumor cell (CTC), Lobectomy, Pulmonary vessel, Ligation, Sequence

## Abstract

**Background:**

The optimal sequence of pulmonary vessel interruption during lung cancer resection remains controversial. This review aimed to elucidate the association of vein-first versus artery-first ligation and survival of the patients.

**Methods:**

We searched PubMed, Web of Science, Scopus, Embase, Cochrane Library and Google Scholar from their inception to September 2021 for published articles that compared vein-first (the pulmonary vein was interrupted first) and artery-first procedure (the pulmonary artery was ligated first) during lung cancer surgery.

**Results:**

Finally, a total of 13 full articles were obtained. First, 7 studies with survival information were included for meta-analyses. As compared with the artery-first ligation, vein-first approach did not decrease the risk of local recurrence (risk ratio [RR] 0.92 in favour of vein-first; 95% confidence interval [CI] 0.61–1.39, p = 0.68) or distant metastasis (RR 0.92; 95% CI 0.30–2.85, p = 0.89); but it was associated with better disease-free survival (RR 0.52; 95% CI 0.37–0.73, p < 0.01) as well as 5-year overall survival (RR 0.60; 95% CI 0.41–0.86, p < 0.01). In addition, the operative time, intraoperative blood loss, total complications, and length of postoperative stay were mainly comparable between the two groups. Second, 7 studies provided the data of tumor cells indicated by different biomarkers and detection methods; and 3 of these reports showed that vein-first ligation decreased the extent of intraoperative tumor dissemination. However, a quantitative meta-analysis was not possible due to the significant heterogeneity.

**Conclusion:**

Vein-first ligation in lung cancer surgery may be associated with improved survival of the patients, which might be ascribed to potentially lower risk of tumor cell dissemination. Well-designed, large-scale trials are warranted to clarify these occasional findings.

## Background

Surgical manipulation during lung cancer resection may dislodge circulating tumor cells (CTCs) into the effluent pulmonary vein (PV) [1]. In theory, blocking the PV of the tumor-bearing lobe first could decrease the risk and amount of iatrogenic tumor dissemination; however, the current studies have drawn controversial conclusions regarding the prognosis of non-small cell lung cancer (NSCLC) patients [2].

In 2015, a review of 7 studies showed that the ligation sequence of the pulmonary vessels did not influence the survival of lung cancer patients [3]. A propensity-score matched analysis in 2019 found that the NSCLC patients who underwent vein-first procedure demonstrated better survival compared to those in the artery-first group. In addition, the CTCs in peripheral blood were significantly decreased in vein-first patients than the control [4]. Nevertheless, vein-first ligation was correlated with better survival only in patients with stage I and II diseases [4]; whereas another study found that vein-first procedure only benefited stage I patients [5].

To the best of our knowledge, it is more convenient for the surgeons to ligate artery first in uniportal thoracoscopic lung resection; meanwhile, some surgeons advocate artery-first because it might decrease the amount of blood loss [6]. Herein an updated systemic review was conducted to qualitatively elucidate the role of vein-first versus artery-first interruption in lung cancer surgery.

## Methods

The Preferred Reporting Items for Systematic Reviews and Meta-Analyses (PRISMA) statement was used as reported [7]. This review was approved for publication by the Ethics Committee and Institutional Review Board of Xuzhou Central Hospital.

### Literature search strategy

We searched PubMed, Web of Science, Scopus, Embase, Europe PMC, Cochrane Library and Google Scholar for studies up to September 2021 based on the population, intervention, comparator, outcome and strategy (PICOS) framework according to the Preferred Reporting Items for Systematic Reviews and Meta-Analyses (PRISMA) Protocol. Key words and MeSH terms in title or abstract including (1) “sequence” or “order” and (2) “ligation” or “sequence” or “interruption” or “resect*” or “dissect*” and (3) (“pulmonary” or “lung”) and (“vessel” or “vasculature” or “vein” or “artery”) and (4) “surgery” and “lung neoplasms/lung cancer” were used. The search strategy was as follows: ((ligation [Title/Abstract]) OR (interruption [Title/Abstract]) OR (resect* [Title/Abstract]) OR (dissect* [Title/Abstract])) AND ((pulmonary [Title] OR lung [Title] OR lobectomy [Title] OR (lung cancer surgery [Title])). No restriction was made regarding the publication language.

### Selection criteria

The selection of studies was based on the titles, abstracts and full papers. Inclusion criteria were as follows: pathological diagnosis of lung cancer; comparative studies examining vein-first versus artery-first procedure during surgery; randomized controlled trials (RCTs), observational (retrospective/prospective cohort and case–control) studies; and studies that reported at least 1 outcome of interest such as the number of CTCs, the biomarkers (such as messenger ribonucleic acid [mRNA]) of tumor cells, postoperative local recurrence, distant metastasis, and survival. From the selected articles, the full texts were reviewed, followed by a decision on their eligibility for inclusion in a meta-analysis. Literature review, meta-analyses, letters to the editor, comments, correspondence, case reports, surgical technique notes, meeting abstracts, duplication publications or second analysis of the same database, unpublished studies, and single-arm reports (without control) were excluded.

### Data collection

For each study, we identified the change of the biomarkers of CTCs or tumor cells in the PV and survival of the patients who underwent vein-first procedure (vein-first group) versus those who firstly ligated or resected the pulmonary artery (artery-first group) during lobectomy for cancer. The related studies were reviewed and the data were extracted by two researchers. Disagreements were resolved by consensus. Then these studies were exported to EndNote (Analytics, Philadelphia, PA, USA) for de-duplication [8].

The level of evidence was assessed by two independent reviewers and categorized per the Oxford Centre for Evidence-Based Medicine 2011 Levels of Evidence [9]. The quality of evidence was graded by two authors independently. Disagreements were resolved by consensus. The primary endpoint was the change of CTCs or tumor cells or their biomarkers in the effluent PV; whereas the secondary endpoints were local recurrence, distant metastasis, disease-free survival (DFS), and overall survival (OS) of the patients after lobectomy with curative intent, respectively.

### Statistical analysis

Risk ratio (RR) with 95% confidence intervals (CI) was calculated for categorical outcomes (local recurrence, distant metastasis, and mortality during the follow up). Study bias was detected using the methods of funnel plots test. Statistical significance was taken as 2-sided p value < 0.05. Meanwhile, the meta-analyses were performed with a random-effect model (rather than fixed-effect model). The meta-analyses were performed using RevMan software 5.3 (The Cochrane Collaboration, Copenhagen, Denmark).

## Results

**Fig. 1:**
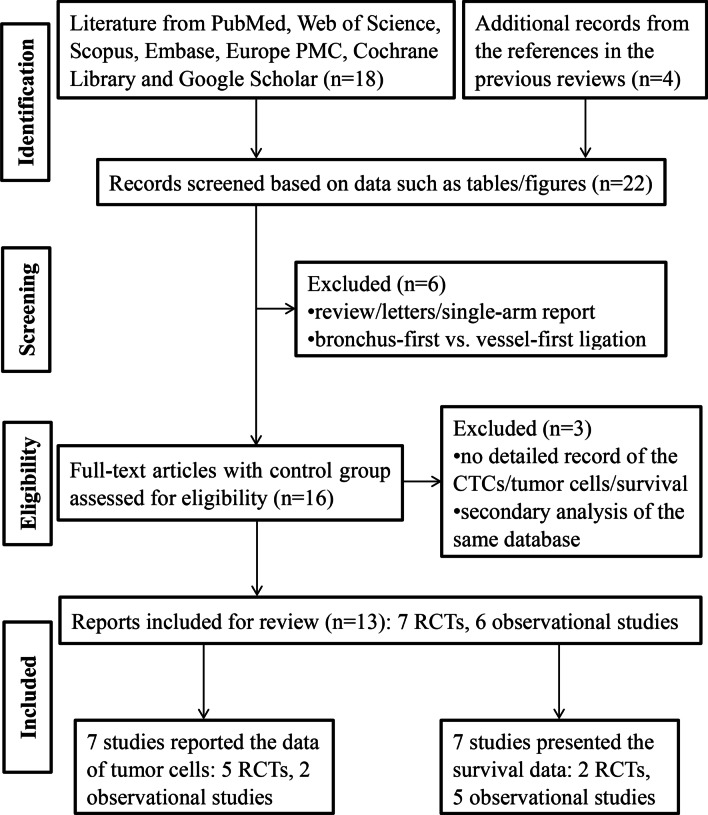
Flow chart

The initial search resulted in 22 studies. Finally, 13 full articles including 7 RCTs and 6 retrospective studies were included for review (Fig. 1). The sample sizes were mainly small (range from 11 to 210 patients in each report); therefore, the evidence level was mainly downgraded.

### The effect of vein-first ligation on survival of the patients

To date, 7 articles (2 RCTs and 5 observational studies) involving 1688 patients had been published, which investigated the role of vein-first procedure in the prognosis of the patients (Table 1) [4, 5, 10–14].Vein-first ligation was performed on 869 patients, while the other 819 patients underwent artery-first surgery. Among them, 4 studies indicated that the interruption sequence of the vessels did not significantly affect the survival; whereas the other 3 studies demonstrated an obvious survival benefit for the patients in vein-first group.

**Table 1:** The oncological results in the 7 included studies (vein-first versus artery-first)

References	Country	Study design	Level of evidence^*^	No. of patients	Tumor staging	Local recurrence	Distant metastasis	5-year DFS	5-year OS	HR (95% CI) of the sequence for survival	Comments
Kozak [10]	Poland	Single-center RCT	2	170/215	I–III, p > 0.05	NR	Total: 69 (17.9%)	NR	92 (54%) vs. 108 (50%), p > 0.05	OS: 0.927 (0.708–1.214) (artery-first as reference)	–
Bai [11]	China	Single-center RCT	3	45/45	I: 39/38; II: 6/7, p > 0.05	3 (6.7%) vs. 2 (4.4%), p > 0.05	8 (17.8%) vs. 6 (13.3%), p > 0.05	29.5 months vs. 28.3 months, p > 0.05	NR	OS: 0.154 (0.251–0.852) (artery-first as reference)	–
Refaely [12]	Israel	Retrospective	4	133/146	I: 77/75; II: 21/29; III: 29/39; IV: 6/3, p > 0.05	68 (51%) vs. 78 (53%), p > 0.05	NR	NR	NR	Not significant	–
Li [13]	China	Retrospective	4	174/93	I: 138/79; II: 36/14, p > 0.05	6 (3.4%) vs. 2 (2.2%), p > 0.05	22 (12.6%) vs. 7 (7.5%), p > 0.05	Not significant	Not significant	Not significant	–
Sumitomo [5]	Japan	Retrospective	4	104/83	I: 91/70; II: 5/8; IIIA: 8/5, p > 0.05	4 (3.8%) vs. 6 (7.2%), p > 0.05	3 (2.9%) vs. 9 (10.8%), p < 0.05	92 (88.2%) vs. 63 (75.7%), p < 0.05	94 (90.9%) vs. 69 (82.7%), p > 0.05	DFS: 2.127 (1.009–4.481) (vein-first as reference)	DFS of stage I (91 vs. 70), p < 0.05; DFS of stage II - IIIA (13 vs. 13), p > 0.05
He [14]	China	Retrospective	4	33/27	I: 14/10; II: 8/9; III: 10/8; IVA: 1/0, p > 0.05	NR	NR	13 (39.40%) vs. 8 (29.6%), p > 0.05	22 (66.7%) vs. 12 (44.4%), p > 0.05	-	OS/PFS of squamous type (10 vs. 8), p < 0.05
Wei [4]	China	Retrospective PSM	4	210/210	I–II	NR	NR	134 (63.6%) vs. 102 (48.4%), p < 0.05	155 (73.6%) vs. 121 (57.6%), p < 0.05	OS: 1.65 (1.07–2.56) (vein-first as reference)	Vein-first better survival in stage I/ II

RCT, randomized controlled trial; NSCLC, non-small cell lung cancer; SCLC, small cell lung cancer; PSM, propensity-score matched analysis; PV, pulmonary vein; OS, overall survival; DFS, disease-free survival; HR, Hazard ratio; CI, confidence interval; NR, not reported

*According to the Oxford Centre for Evidence-Based Medicine 2011 Levels of Evidence

The RCT by Kozak et al. randomized 385 NSCLC patients in the vein-first (n = 170) and artery-first group (n = 215) respectively [10], which demonstrated similar 5-year OS. Refaely et al. retrospectively reviewed 279 NSCLC patients, and both groups (133 cases in vein-first and 146 cases in artery-first) reported similar tumor recurrence [12]. Li et al. also reported similar OS; whereas artery-first procedure could reduce bleeding and postoperative complications [13]. Bai et al. found that vein-first vs. artery-first procedure did not affect the outcomes of early stage NSCLC patients in terms of local recurrence (6.7% vs 4.4%; p > 0.05) and distant metastasis (17.8% vs 13.3%; p > 0.05) [15].

On the contrary, a propensity-matched analysis showed that the vein-first patients demonstrated significantly better 5-year OS (73.6% vs 57.6%; p < 0.01), DFS (63.6% vs 48.4%; p < 0.01) and lung cancer-specific survival (76.4% vs 59.9%; p < 0.01) than the control [4]. In addition, a retrospective study showed that the DFS of the patients in vein-first and non-vein-first groups was 6.7% (7/104) and 18.1% (15/83), respectively (p < 0.05) [5]. Moreover, a retrospective study of 60 NSCLC patients (33 in vein-first and 27 in artery-first group) reported similar OS (p > 0.05); however, subgroup analysis revealed that vein-first procedure delivered better survival in squamous cell carcinoma patients [14].

Furthermore, Wei et al. reported that vein-first procedure was correlated with better survival in stage I and stage II patients but not stage III cases [4]. Sumitomo et al. also indicated that vein-first ligation provided better survival for the patients in stage I but not stage II or IIIA diseases [5].

In addition, the perioperative characteristics of the patients (vein-first versus artery-first) were shown in Table 2. Because only 2 RCTs with quite limited sample have been reported to date, we only used Level of Evidence instead of risk of bias to classify the studies. Operative time, intraoperative blood loss, total complications, and postoperative hospital stay were mainly comparable between the two groups.

**Table 2:** Perioperative characteristics of the patients (vein-first versus artery-first)

References	Study design	No. of patients	Operation time, min	Blood loss, mL	Total complications, n(%)	Postoperative hospital stay, d
Kozak [10]	Single-center RCT	170/215	Similar, p > 0.05	Similar, p > 0.05	Similar, p > 0.05	Similar, p > 0.05
Bai [11]	Single-center RCT	45/45	(187.9 ± 28.9) vs. (177.2 ± 27.3), p > 0.05	(158.3 ± 22.6) vs. (105.8 ± 21.9), p < 0.05	13(28.9) vs. 15(33.3), p > 0.05	(9.8 ± 1.5) vs. (9.3 ± 1.3), p > 0.05
Refaely [12]	Retrospective	133/146	NR	NR	Similar, p > 0.05	(8.9 ± 4.9) vs. (9.5 ± 5.0), p > 0.05
Li [13]	Retrospective	174/93	(186.2 ± 53.6) vs. (167.0 ± 45.2), p > 0.05	(148.3 ± 142.9) vs. (105.1 ± 97.5), p < 0.05	44 (25.3) vs. 23 (24.7), p > 0.05	(9.5 ± 9.9) vs. (8.0 ± 4.0), p > 0.05
Sumitomo [5]	Retrospective	104/83	(227.5 ± 60.2) vs. (222.5 ± 47.2), p > 0.05	(101.9 ± 148.6) vs. (125.6 ± 123.8), p > 0.05	NR	NR
He [14]	Retrospective	33/27	NR	NR	NR	NR
Wei [4]	Retrospective PSM	210/210	119 (100–150) vs. 123 (110–155), p > 0.05	50 (20–55) vs. 50 (20–95), p > 0.05	5 (13.2) vs. 5 (12.5), p > 0.05	6 (5–8) vs. 6 (5–7), p > 0.05

RCT, randomized controlled trial; NSCLC, PSM, propensity-score matched analysis; NR, not reported

### Quantitative data synthesis

**Fig. 2:**
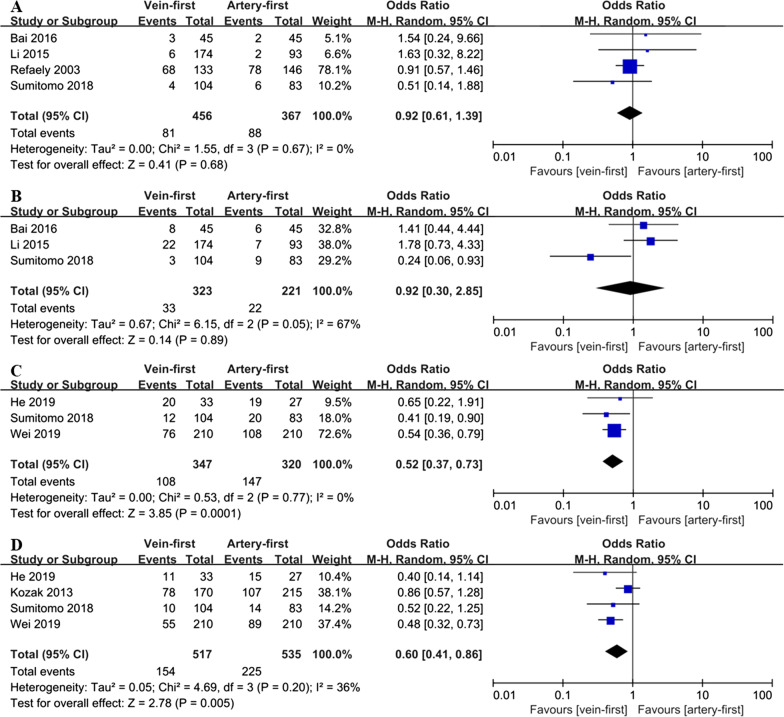
Forest plots for the comparisons of oncological outcomes. **A** Local recurrence; **B** distant metastasis; **C** disease-free survival; **D** overall survival

Only 7 reports in Table 3 were possible for meta-analyses. The forest plots for the comparisons of local recurrence, distant metastasis, DFS and OS between the groups were generated respectively (Fig. 2).

**Table 3:** The change of tumor cells in the effluent pulmonary veins after lobectomy (vein-first versus artery-first)

References	Country	Study design	Level of evidence^*^	No. of patients	Tumor staging	Change of CTCs after surgery in the effluent PV	Indicators of tumor cells in PV	HR (95% CI) of the sequence for CTCs
Wei [4]	China	Multicenter RCT (NCT03436329)	3	NSCLC: 38/40	I: 22/21; II: 10/10; III: 9/12; IV: 2/0, p > 0.05	Incremental changes of CTCs: 31.6% (12/38) vs. 65.0% (26/40), p < 0.05	NR	4.03 (1.53–10.63), p, 0.005
Kurusu [15]	Japan	Single-center RCT	4	NSCLC: 15/15 SCLC: 3/3	I: 14/15; IIIA: 4/3, p > 0.05	NR	Positive CEA mRNA in initially negative samples: 42.9% (3/7) vs. 85.7% (6/7), p > 0.05	NR
Ge [16]	China	Single-center RCT	4	NSCLC: 12/11	I: 6/3; II: 3/4; III: 3/4, p > 0.05	NR	Postoperative CEA mRNA was increased similarly, p > 0.05	NR
Ai [17]	China	Single-center RCT	4	NSCLC: 14/12	I: 3/3; II: 2/2; III: 9/7	NR	The Pin 1 mRNA was changed similarly, p > 0.05	NR
Song [18]	China	Single-center RCT	4	NSCLC: 15/15	I/II/III: 9/13/8	NR	Serum cytokeratin 19 (CK19)/adhesion molecule CD44v6 mRNA was changed significantly after surgery in artery-first group, but not vein-first group, p < 0.05	NR
Duan [1]	China	Retrospective	4	NSCLC: 19/14	I/II/III: 21/6/6	The increases of CTC after surgery was higher in the vein-first group (+ 11 vs. + 4.5), p < 0.05	NR	NR
Hashimoto [19]	Japan	Retrospective	4	NSCLC: 9/21	I/II/III/IV: 17/8/3/2	The increase of CTCs after surgery was similar between the groups (+ 33 vs. + 56), p > 0.05	NR	NR

PV, pulmonary vein; CTCs, Circulating tumor cells; NSCLC, non-small cell lung cancer; SCLC, small cell lung cancer; Pin 1, peptidyl-prolyl cis–trans isomerase NIMA-interacting 1; NR, not reported

*According to the Oxford Centre for Evidence-Based Medicine 2011 Levels of Evidence

For the 4 reports with a local recurrence rate, the heterogeneity was as follows: I^2^ = 0%. No significant difference in terms of local recurrence was noted (RR 0.92 in favour of vein-first ligation; 95% confidence interval [CI] 0.61–1.39, p = 0.68). Moreover, for the 3 reports with distant metastasis data, the heterogeneity was as follows: I^2^ = 67%. No significant difference regarding distant metastasis was observed between the two groups (RR 0.92 in favour of vein-first ligation; 95% CI 0.30–2.85, p = 0.89).

Three reports presented detailed DFS information, and the heterogeneity was as follows: I^2^ = 40%. Vein-first group demonstrated lower risk of mortality compared to the control (RR 0.52 in favour of vein-first ligation; 95% CI 0.37–0.73, p < 0.001). Similarly, for the 4 reports with OS data, the heterogeneity was as follows: I^2^ = 63%. Vein-first group demonstrated significantly better 5-year overall survival compared to the counterpart (RR 0.60; 95% CI 0.41–0.86, p = 0.005).

### Sensitivity analysis

**Fig. 3:**
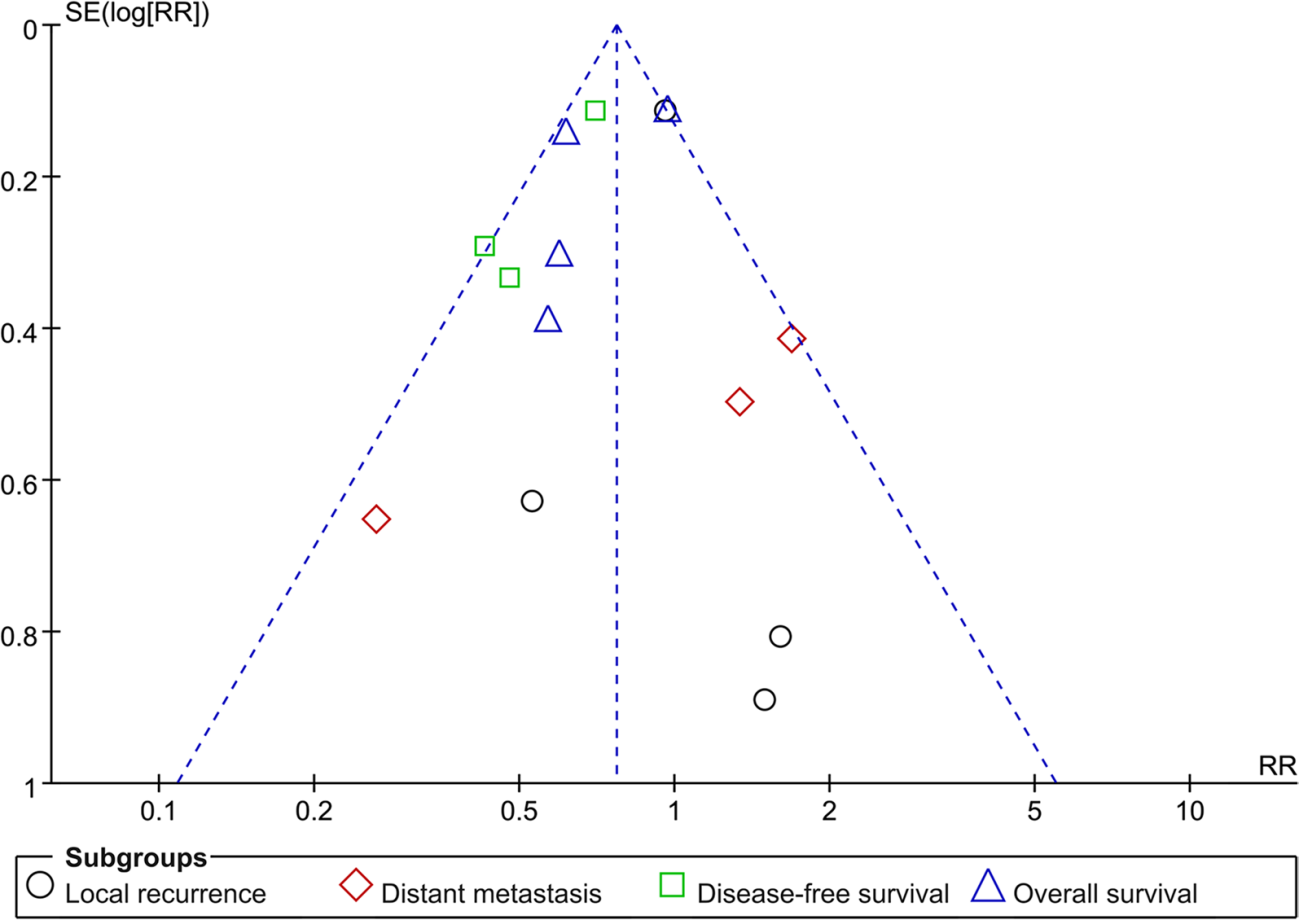
Funnel plot of publication biases of the 7 studies in the meta-analyses

We combined different study types in the above meta-analyses. Thus, a sensitivity analysis was conducted. Actually, the available data, especially prospective studies regarding DFS and OS, are quite limited to provide an adequately powered meta-analysis. Thus, a sensitivity analysis is not significantly helpful to strengthen the quality of this meta-analysis. When the 2 RCTs were excluded from the meta-analysis one by one (Kozak, 2013; Bai, 2016) [10, 11], the results in favor of vine-first resection were not changed. In detail, both groups showed similar local recurrence and distant metastasis rate (p > 0.05, respectively); whereas the vein-first patients demonstrated better 5-year OS compared to artery-first group (RR 0.61; 95% CI 0.48–0.77, p < 0.001). Furthermore, the funnel plot in the meta-analysis suggested publication bias (Fig. 3).

### The effect of vein-first ligation on CTCs

Seven studies presented the change of tumor cells in the effluent PV after lung cancer surgery (Table 3), including 5 RCTs and 2 observational studies [1, 4, 15–19]. Three of them indicated that vein-first ligation was associated with a lower risk of intraoperative tumor dissemination; whereas the others recorded similar outcomes. However, a quantitative meta-analysis regarding CTCs was not possible because these studies used different tumor biomarkers and calculation methods.

A multicenter RCT (NCT03436329) showed an incremental change of CTCs in 26 of 40 patients (65.0%) in the artery-first group and 12 of 38 (31.6%) in the vein-first group (p < 0.01) after surgery for NSCLC [4]. Kurusu et al. examined the presence of CTCs as reflected by carcinoembryonic antigen (CEA) mRNA in 30 NSCLC patients [15]. Of the 14 initially negative samples (7 in each group), 9 samples became positive during the operation, and such conversion was more common with artery-first (6 patients, 85.7%) than vein-first procedure (3 patients, 42.9%). In addition, exploiting cytokeratin 19 and CEA mRNA as markers of malignant cells, Ge et al. collected 23 NSCLC patients [16], which showed that vein-first procedure may partly prevent release of tumor cells into bloodstream. Moreover, Song et al. randomized NSCLC patients into artery-first and vein-first group (15 cases in each) [18]. The expression of cytokeratin 19 and adhesion molecule CD44v6 mRNA as biomarkers of lung cancer micrometastasis in the late period of surgery were higher than those in the early period in artery-first group (p < 0.05); whereas neither the cytokeratin 19 nor CD44v6 after surgery in the vein-first group exhibited significant change versus those before surgery (p > 0.05). The authors therefore concluded that vein-first ligation help lower the risk of manipulation-related micrometastasis. Similarly, a prospective study of 33 patients showed that the number of CTCs was 3.36 before PV interruption; whereas it increased to be 14.88 after lobectomy for lung cancer [1]. Therefore, surgical manipulation may dislodge tumor cells into the PV but vein-first procedure may decrease the CTCs entry into the circulation.

On the other hand, Hashimoto et al. reported that the increase of CTCs in the PV was not significantly associated the sequence of vessel interruption [19].

## Discussion

The present meta-analyses including 7 studies published from 1998 to 2021 tried to compare the survival using RR because only 2 hazard ratios was available. In the study, no difference between the vein-first and artery-first groups was indicated in terms of postoperative recurrence and distant metastasis, but the patients underwent vein-first ligation procedure showed obviously lower 5-year overall mortality as compared to the control group. We provide updated evidence supporting vein-first surgery for improved survival. In theory, the potentially improved prognosis might be ascribed to lower surgery-related tumor cell dissemination. Nevertheless, the current studies regarding the risk of CTCs dissemination is still insufficient for meta-analysis.

Only a review has so far been published [3]. Toufektzian et al. qualitatively summarized the results of 6 prospective and 1 retrospective studies; and thy found that the sequence of pulmonary vessels ligation did not seem to influence the oncological outcomes of lung cancer patients. Our study included several newly presented reports, and conducted the first meta-analysis of survival associated with the sequence of vessels ligation in lung cancer surgery. Due to publication bias, small samples, heterogeneity and the inherent limitations of meta-analysis involving observational studies, our findings should be interpreted with caution.

The increase of CTCs after surgical manipulation might explain the distant metastases after tumor resection [20]. The detection methods of CTCs and the type of mRNA were inconsistent in the present review; therefore, standardized criteria for the collection of blood samples and detection of the CTCs as well as the tumor cells in PV should be considered in further trials. Moreover, surgeons’ experience was correlated with the operation time before PV interruption, which might explain the distinct prognosis of lung cancer patients treated by different surgeons [21]. As a result, it is probably indisputable that oncological surgery should be performed by experienced surgeons to avoid excessive manipulation of the tumor-bearing lobe before the interruption of the effluent PV.

Similarly, the operation time before PV ligation (later or earlier than artery interruption) may affect the quantity of tumor cells released into the circulation. However, to date we find only 1 registered trial (ChiCTR1800016879) evaluating the effect of different timing before PV ligation on the prognosis of the lung cancer patients. The registered trials comparing vein-first and artery-first ligation in lung cancer surgery were listed in Table 4. A definite conclusion might be drawn from the forthcoming researches.

**Table 4:** The registered trials of vein-first versus artery-first ligation in lung cancer surgery

Identifier	Year	Neoadjuvant chemotherapy or radiotherapy	Staging	Surgical procedure	Study design	Estimated enrollment	Major outcomes	Status	Country
NCT00341380	2006	Not mentioned	Stage I ~ II	Resection of non-small cell lung tumor	Prospective cohort study	41	Postoperative metastases/recurrence	Completed	America
NCT03645252 (CTC-01)	2018	None	cT1 ~ 3N0M0	Lobectomy/bi-lobectomy	Randomized pilot study	30	The change of CTC in pulmonary vein; DFS; OS	Not yet recruiting	Canada
NCT03436329	2018	None	Stage I–IV eligible for surgery	Lobectomy	Multi-centre randomized controlled trial	60	CTC level before cutting the skin and after closing the chest; 3-year PFS; complications	Not yet recruiting	China
ChiCTR1800016879	2018	None	cT1a-2aN0M0	Lobectomy	Non-randomized cohort study	60	The change of CTC level	Recruiting	China

CTC, circulating tumor cell; PFS, progression-free survival; DFS, disease-free survival; OS, overall survival

Moreover, the disadvantages of vein-first lung resection should not be neglected. Vein-first procedure was reported to be associated with more intraoperative blood loss as compared with artery-first surgeries [13]. It might be difficult to separate the PV first before the removal of calcified lymph nodes around the PV. Moreover, not all the branches of the PV with anatomic variants could be interrupted simultaneously and quickly before artery ligation. Considering the safety of the patients, artery-first or mixed procedure, instead of vein-first ligation, might be the optimal choice when the PV is deeply located.

The available data of perioperative complications were listed in Table 2, which showed that the operative time, intraoperative estimated blood loss, total complications, and length of postoperative stay were mainly comparable between the two groups. Detailed information of the events is not available and truly limited, so additional meta-analyses are not possible. Further trials are necessary to clarify the actual role of vein-first ligation in surgery-related complications such as bleeding and conversion to open thoracotomy during thoracoscopic lung cancer surgery. Because only 2 RCTs regarding oncological results with quite limited samples have been reported to date, we only used Level of Evidence, instead of Cochrane risk-of-bias tool, to classify the quality of the retrieved studies.

Lastly, we acknowledge several significant limitations to this review. First, 5 of the 7 reports in the meta-analyses indicated low quality of evidence and great risk of bias due to small samples and retrospective nature. The articles of different study types (RCTs and non-RCT reports) were pooled both together and separately for sensitivity analysis. The estimates from observational studies might be overestimated due to selection bias, which might result in misleading information. Meanwhile, the potential items correlated with survival of the patients including but not limited to neoadjuvant treatment, the operation time before PV interruption, the stations and total number of dissected lymph nodes, and postoperative multimodal therapeutic regimens, which were not reported in detail in most of the studies. Second, different surgical preference, experience, and learning curve of clinicians might also affect the outcomes. Third, unpublished data and relevant articles in non-English and non-Chinese databases may be neglected. Furthermore, the detailed information of perioperative complications and quality of life are not definitely available from the retrieved studies. Further well-designed trials are warranted to clarify the role of vein-first procedure in manipulation-related complications including bleeding and conversion to open surgery during lung cancer surgery. In addition, the following issues such as open vs. minimal-invasive surgery, extent of surgery (lobectomy vs. pneumonectomy vs. segmentectomy), length-of-stay in hospital and intensive care unit (ICU), perioperative chemotherapy and immunotherapy, type and stage of cancer should also be incorporated and compared to further define the definite role of vein-first procedure in lung cancer resection. Thus, the actual benefit of vein-first ligation for patients undergoing lung cancer surgery largely remains unanswered.

## Conclusions

Vein-first ligation in lung cancer surgery may be associated with improved prognosis and potentially decreased risk of manipulation-related tumor dissemination. Well-designed, large-scale trials are warranted to verify these findings before a definite recommendation could be drawn.

## Data Availability

All data generated or analyzed during this study are included in this published article.

## Abbreviations

NSCLC: Non-small cell lung cancer
CTC: Circulating tumor cell
PA: Pulmonary artery
PV: Pulmonary vein
DFS: Disease-free survival
OS: Overall survival
CI: Confidence interval
RR: Risk ratio
RCTs: Randomized controlled trials
CEA: Carcinoembryonic antigen
